# Exit of *Plasmodium* Sporozoites from Oocysts Is an Active Process That Involves the Circumsporozoite Protein

**DOI:** 10.1371/journal.ppat.0010009

**Published:** 2005-09-30

**Authors:** Qian Wang, Hisashi Fujioka, Victor Nussenzweig

**Affiliations:** 1 Department of Pathology, Michael Heidelberger Division, New York University School of Medicine, New York, New York, United States of America; 2 Institute of Pathology, Case Western Reserve University, Cleveland, Ohio, United States of America; Northwestern University Medical School, United States of America

## Abstract

*Plasmodium* sporozoites develop within oocysts residing in the mosquito midgut. Mature sporozoites exit the oocysts, enter the hemolymph, and invade the salivary glands. The circumsporozoite (CS) protein is the major surface protein of salivary gland and oocyst sporozoites. It is also found on the oocyst plasma membrane and on the inner surface of the oocyst capsule. CS protein contains a conserved motif of positively charged amino acids: region II-plus, which has been implicated in the initial stages of sporozoite invasion of hepatocytes. We investigated the function of region II-plus by generating mutant parasites in which the region had been substituted with alanines. Mutant parasites produced normal numbers of sporozoites in the oocysts, but the sporozoites were unable to exit the oocysts. In in vitro as well, there was a profound delay, upon trypsin treatment, in the release of mutant sporozoites from oocysts. We conclude that the exit of sporozoites from oocysts is an active process that involves the region II-plus of CS protein. In addition, the mutant sporozoites were not infective to young rats. These findings provide a new target for developing reagents that interfere with the transmission of malaria.

## Synopsis

Malaria affects hundreds of millions of people, and kills at least 1 million children per year. The infective stages of the malaria parasites, named “sporozoites,” are found in the salivary gland of *Anopheles* mosquitoes, and are injected along with the saliva during blood feeding. From the skin, sporozoites enter the blood circulation and invade liver cells where the parasites multiply. When they exit the liver, these parasites infect blood cells and can cause severe symptoms. If ingested by mosquitoes, the blood-stage parasites continue their lifecycle in the insect stomach. Thousands of sporozoites are formed within a cyst-like structure (oocyst). The sporozoites come out of the oocyst and infect the salivary gland, where they remain until injected back into humans. Malaria parasites are increasingly resistant to drugs, mosquitoes are difficult to eliminate, and effective vaccines are not yet available. New tools to combat malaria are urgently needed. One exciting approach, although the application is in the distant future, is to release in endemic areas genetically modified mosquitoes that are resistant to parasite growth. This paper provides a new target for generating these “transmission-block” mosquitoes and shows that the exit of sporozoites from the oocysts is an active process that requires the enzymatic digestion of components of the oocyst wall. If these enzymes are inhibited in transgenic mosquitoes, sporozoites will never reach the salivary gland.

## Introduction

The *Plasmodium* life cycle in the *Anopheles* mosquitoes begins with the ingestion of a blood meal containing *Plasmodium* gametocytes. After fertilization of the resulting gametes, the zygotes transform into motile ookinetes that traverse the midgut epithelium, reach the basal lamina, and then transform into oocysts. The young oocyst, surrounded by a capsule and by the basal lamina, undergoes multiple mitotic nuclear divisions and progressively enlarges in size without cytokinesis. At the same time, the cytoplasm is subdivided by membrane clefts forming structures named “sporoblasts.” Later, uninucleate sporozoites bud from the sporoblast membrane. The mature oocyst is about 50 μm in diameter and contains thousands of sporozoites. Sporozoites leave the oocysts asynchronously, enter the hemolymph, and then invade the salivary glands where they remain until they are injected, with the saliva, into the skin of the mammalian host [1,2].

In order to reach the flowing hemolymph, sporozoites must traverse two physical barriers: the oocyst capsule and the mosquito basal lamina. Because oocyst sporozoites display limited movement [3], their egress from oocysts is generally thought to be a passive process. Early ultrastructural observations reveal the presence of small openings in the capsule of mature oocysts and the basal lamina. Occasionally, sporozoites are found “penetrating” these openings and entering the hemolymph [4]. The oocyst capsule contains laminin of mosquito origin and displays *trans-*glutaminase activity probably of parasite origin [5,6]. In addition, the inner surface of the capsule is covered with the *Plasmodium* circumsporozoite (CS) protein [7–9].

The development of sporozoites in oocysts is CS dependent. When the *CS* gene is deleted, the oocysts are devoid of mature parasites [10]. To investigate the mechanisms leading to this developmental arrest, we have generated *Plasmodium berghei* parasites bearing different mutations in the *CS* coding region. In one of the *P. berghei CS* mutants, we substituted the positively charged amino acids of the conserved region II-plus with alanines. Region II-plus is located at the 5′ end of the thrombospondin type 1 repeat (TSR) domain of CS protein. Several in vitro observations strongly suggest that region II-plus participates in the initial steps of sporozoite attachment and invasion of the host's hepatocytes via interaction with heparan sulfate proteoglycan (HSPGs) on the host cells [11,12]. Here we show for the first time that the mutation in region II-plus of CS protein prevents the exit of sporozoites from oocysts and progression of the *Plasmodium* lifecycle. In addition, the mutant sporozoites are unable to infect rats.

## Results/Discussion

### Construction of CS-RIImut and CS-WT

**Figure 1:**
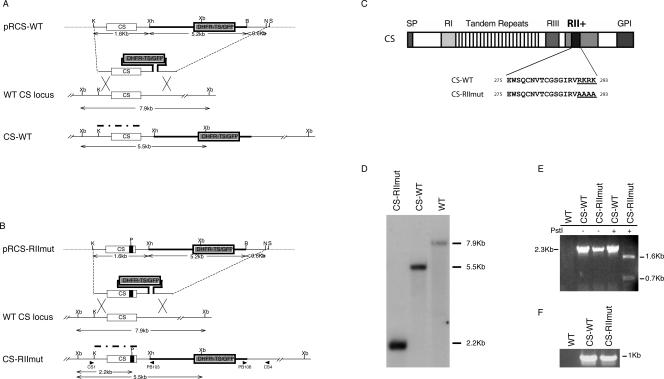
Gene Targeting at the *CS* Locus of *P. berghei* **(**A and B**)** Replacement plasmid pRCS-WT and pRCS-RIImut, wild-type (WT) *CS* locus and recombinant locus. ORFs are symbolized by boxes. Small black box in the *CS* ORF indicates the mutation in region II-plus (R290A, K291A, R292A, and K293A). A PstI site is introduced in the mutation site to differentiate CS-RIImut from CS-WT. Thick lines indicate 5′- and 3′-UTRs of *DHFR-TS;* thin lines, 5′- and 3′UTRs of *CS;* dashed lines, plasmid vector sequence. B, BamHI; K, KpnI; N, NotI; P, PstI; S, SacI; Xb, XbaI; Xh, XhoI. Recombinant CS-WT or CS-RIImut was generated by double crossover occurring between the CS sequences in the KpnI-SacI fragment of plasmid pRCS-WT or pRCS-RIImut and their homologous sequence in the wild-type *CS* genomic locus. The CS probe used in the genomic Southern hybridization is symbolized by a thick dash-dot line. Restriction fragments of the wild type and of the expected recombinants are shown below the corresponding locus. Locations of primers used for PCR are indicated in (B). (C) Schematic structure of the CS protein and sequences of the region II-plus of wild-type and mutant CS. (D) Genomic Southern hybridization of the wild-type *P. berghei* strain (WT), the recombinant lines, control (CS-WT), and mutant (CS-RIImut) parasites upon digestion with XbaI and PstI, using a CS probe. (E) PCR amplification with primers CS1 and PB103 of the expressed CS protein at the 5′ recombinant locus in CS-RIImut and CS-WT. The WT is used as a negative control (lack of recombination events). The amplicons (2.3 kb) were subjected to PstI restriction enzyme digestion (two fragments released, 1.6 and 0.7 kb) to examine the presence of mutation in CS-RIImut, which is absent in CS-WT. (F) PCR amplification of the 3′ recombinant locus using primers PB106 and CS4.

A *P. berghei* clone with mutated region II-plus of CS protein (R290A, K291A, R292A, and K293A) was obtained by homologous recombination. In order to avoid any potential defects in the locus associated with the recombination event, a control clone, CS-WT, which produces wild-type CS protein, was generated by the same method (pRCS-WT and pRCS-RIImut, Figure 1A and 1B). A PstI site was introduced in pRCS-RIImut in order to detect the presence of mutations by PCR and Southern blot analysis [13]. The schematic structure of CS and the sequence of region II-plus of wild-type and mutant CS are shown in Figure 1C.

Genomic DNA from WT, CS-WT, and CS-RIImut were digested with XbaI and PstI, and subjected to Southern blot hybridization. WT displays a 7.9-kb band, whereas CS-WT displays a 5.5-kb band and CS-RIImut a 2.2-kb band, indicating that the CS-RIImut and CS-WT have a correct recombination locus (Figure 1D). This was confirmed by PCR amplification specific for recombinants (Figure 1E and 1F), subsequent PstI digestion (Figure 1E), and by sequencing of PCR products. The sequences of coding regions of wild-type and mutant *CS* are as expected.

We cannot exclude the possibility that the substitution of the four positively charged amino acids of region II-plus led to secondary changes in the structure of CS protein.

### CS-RIImut Sporozoites Do Not Exit from Oocysts

**Figure 2:**
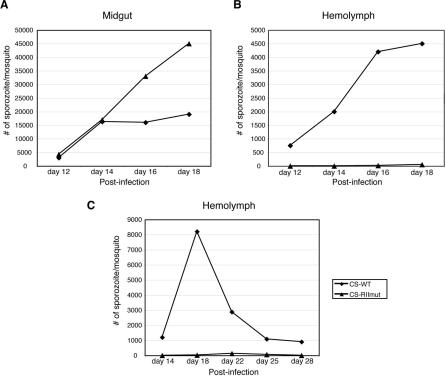
CS-RIImut Oocyst Sporozoites Are Not Released from the Midguts Represented in each graph are the mean numbers of sporozoites per mosquito at different days PI. Each number is calculated based on an average of 20 mosquitoes. (A) Numbers of oocyst sporozoites per mosquito from CS-WT– and CS-RIImut–infected mosquitoes. In CS-RIImut, the number of sporozoites increases progressively until day 18 PI, whereas in CS-WT a plateau is reached at day 14. (B) Numbers of sporozoites in the hemolymph from CS-WT– and CS-RIImut–infected mosquitoes from the same lot as in (A). CS-WT oocysts release sporozoites into the hemolymph from day 12 to day 18 PI. The peak is around day 18 PI. In contrast, the hemolymph from CS-RIImut contains very few sporozoites. (C) In another feeding experiment, the number of hemolymph sporozoites from CS-WT and CS-RIImut infected mosquitoes were calculated from day 14 to day 28 PI. CS-WT releases sporozoites into the hemolymph up to day 28 PI, whereas CS-RIImut does not.

Groups of *Anopheles stephensi* mosquitoes were infected with CS-WT, a clone of wild-type *P. berghei* NK65 strain (WT), and two independent clones of CS-RIImut. CS-WT and WT are identical; therefore, CS-WT was used as a wild type control in all experiments. The numbers of oocysts and oocyst sporozoites at 14 d after blood meal (post-infection [PI]) were very similar in CS-WT and in the two mutant clones (Table 1). However, profound differences were observed at later time points. At day 16 and 18 PI, mutant infected mosquitoes contained many more oocyst sporozoites compared to wild-type infected ones (Figure 2A). In two other independent feeding experiments, similar results were obtained at day 16 PI: 50,000 and 96,000 oocyst sporozoites/mosquito for CS-RIImut, versus 35,000 and 75,000 oocyst sporozoites/mosquito for CS-WT, respectively. By contrast, the hemolymph of mosquitoes infected with CS-WT parasites contained many more sporozoites compared to mosquitoes infected with mutant parasites. CS-WT sporozoites entered the hemolymph beginning at day 12 PI, and the peak numbers were reached around day 18 PI. In contrast, even at 28 days PI, only minimal numbers of CS-RIImut sporozoites were found in the hemolymph (Figure 2B and 2C). Thus, the observed increase in the numbers of CS-RIImut oocyst sporozoites between days 14 and 18 is most likely a consequence of their inability to be released in to the hemolymph.

**Table 1:**
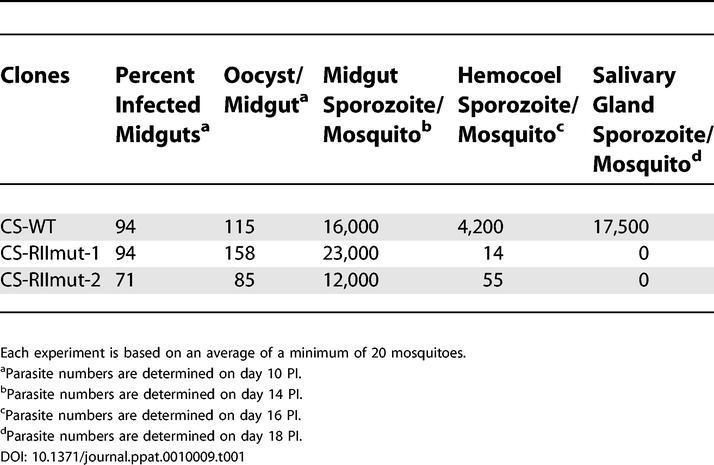
Parasite Development in Mosquitoes

### CS-RIImut Sporozoites Display Normal Morphology and Motility

**Figure 3:**
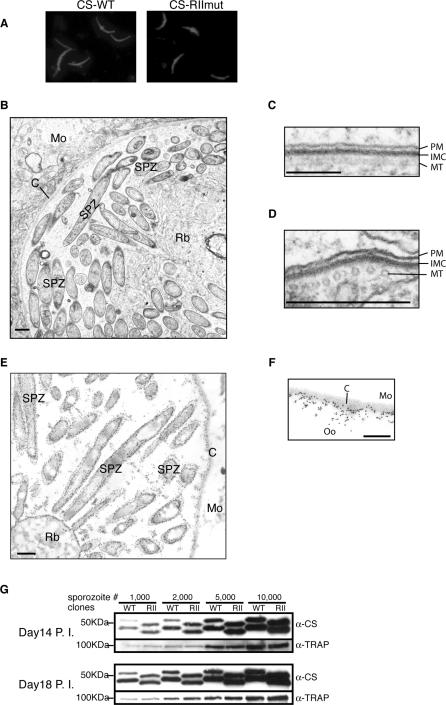
Ultrastructure and CS Localization of CS-WT and CS-RIImut (A) Immunofluorescence labeling of oocyst sporozoites from CS-WT and CS-RIImut at day 18 PI. Sporozoites were stained with anti-CS antibody and detected by FITC-conjugated anti-IgG antibodies, without prior permeabilization. (B) Transmission electron micrographs showing a CS-RIImut oocyst at day 14 PI. The oocyst is surrounded by a capsule and the mosquito basal lamina, and contains sections of sporozoites with normal morphology. Fully developed sporozoites are found within the CS-RIImut oocyst. Sporozoites have homogenous size and morphology. Scale bar represents 1 μm. (C) Longitudinal section of the CS-RIImut sporozoite pellicle shows the plasma membrane, inner membrane complex, and an associated microtubule. Scale bar represents 0.5 μm. (D) Cross-section of a CS-RIImut sporozoite showing the trimembrane pellicle and subpellicular microtubules. Scale bar represents 0.5 μm. (E) Immuno-electron micrographs showing CS localization in the CS-RIImut oocyst. CS protein is predominantly found on the surface of sporozoites and the residual body, and on the inner surface of the capsule. Scale bar represents 1 μm. (F) Enlarged picture of the CS-RIImut oocyst capsule. CS protein is detected on the inner surface of the capsule. Scale bar represents 1 μm. (G) Western blot analysis of extracts from CS-WT (WT) and CS-RIImut (RII) oocyst sporozoites. Numbers of sporozoites loaded are indicated on the top of each lane. Sporozoites were collected from oocysts in mosquito midguts at days 14 and 18 PI. C, capsule; IMC, inner membrane complex; Mo, mosquito tissue; MT, microtubule; Oo, oocyst; PM, plasma membrane; Rb, residual body; SPZ, sporozoite.

The morphology of CS-RIImut sporozoites was analyzed by immunofluorescence assays (Figure 3A), transmission electron microscopy, and immuno-electron microscopy using monoclonal antibodies (3D11) to the repeats of the CS protein. (Figure 3B–3F). CS-RIImut did not display any abnormalities in the sporozoite morphology during development (Figure 3B). The detailed structure of the mutant sporozoite surface is shown Figure 3C and 3D. The structures of the trimembrane pellicle (plasma membrane and inner membrane complex) and subpellicular microtubules are indistinguishable from those of wild type. Patterns of CS protein labeling in CS-WT and CS-RIImut sporozoites were indistinguishable. In the mutants, very similar to wild type, CS protein was detected on the surface of budding or fully developed sporozoites (Figure 3A and 3E), and on the inner surface of the capsule (Figure 3E and 3F) [2,14]. To compare the amounts of CS protein in mutant and CS-WT parasites, extracts of CS-WT and CS-RIImut oocyst sporozoites were analyzed by Western blot (Figure 3G). The intensity of both precursor (54 kDa) and mature (44 kDa) forms of CS [15], was very similar in the WT and the mutant. The two bands appear slightly smaller in the mutant as a result of the replacement of four basic residues (R290, K291, R292, and K293) with alanines. As a control we analyzed levels of TRAP (thrombospondin-related anonymous protein), another sporozoite surface protein [16], and found that it was not affected in the *CS* mutant (Figure 3G). We conclude that the recombination event and mutations did not grossly affect CS protein expression or stability.

It could be argued that the sporozoite exit from oocysts requires sporozoite motility and that the motility is impaired in the mutants. In fact, previous studies have shown that sporozoite motility is neither required nor does it ensure the exit of sporozoites from oocysts [14,17]. In contrast to the circular gliding observed in salivary gland sporozoites, movements of oocyst sporozoites are mostly limited to stretching and back-and-forth gliding [3]. We observed that approximately 3% CS-RIImut oocyst sporozoites display discontinuous gliding motility, and approximately 10%–15% display stretching and bending. The numbers are very similar to those of CS-WT.

### CS-RIImut Oocysts Are More Resistant to Proteolytic Activity

Although CS-WT and CS-RIImut are morphologically indistinguishable, develop equally well in mosquitoes, move similarly, and contain equal levels of CS protein, sporozoite egress from mutant oocyst is profoundly defective. Little is known of the process of sporozoite exit from oocysts, but some information can be obtained from the erythrocytic stages of the parasite. During development in the red blood cells, malaria parasites reside inside a parasitophorous vacuole. Merozoite egress requires the rupture of the parasitophorous vacuole and the membrane of the red blood cells. Release of merozoites from infected erythrocytes requires proteases and is inhibited by inhibitors of proteolytic enzymes [18–20]. *Plasmodium falciparum* falcipain-2 (a cysteine protease) cleaves erythrocyte membrane skeletal proteins at late stages of parasite development [21], facilitating the merozoite egress.

**Figure 4:**
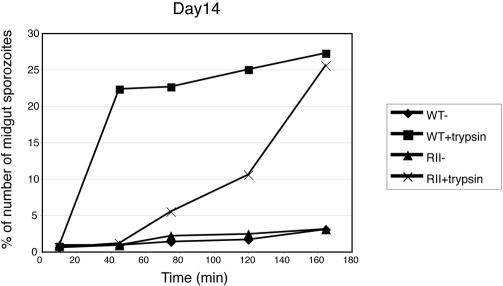
In Vitro Oocyst Sporozoite Release Assay Release of oocyst sporozoites at 25 °C in vitro in the presence of trypsin (50 μg/ml) at day 14 PI. *Y*-axis represents released sporozoites as a percentage of total oocyst sporozoites. *X*-axis indicates the time point when the samples are collected. In the absence of the trypsin, very few sporozoites are released from the midguts. In the presence of the trypsin, oocyst sporozoites from both CS-WT (WT) and CS-RIImut (RII) are released in a time-dependent manner. Compared with CS-WT, CS-RIImut sporozoites are released more slowly.

To examine the possible role of a proteolytic event in the release of sporozoites from oocysts, we treated isolated midguts from CS-WT– or CS-RIImut–infected mosquitoes (14 days PI) with trypsin and measured the number of released sporozoites (Figure 4). The lower temperature (25 °C) was chosen to mimic natural conditions of oocyst development in the mosquito midguts. In the absence of trypsin, very few sporozoites were released from either CS-WT– or CS-RIImut–infected midguts even after 3 h of incubation. Treatment with trypsin for 40 min leads to a significant increase in the number of sporozoites released from CS-WT, but not from CS-RIImut. Release of sporozoites from CS-RIImut oocysts was achieved only after extended treatment with trypsin. In Figure 4 we show the release at 14 d PI, but identical results were observed with 18-d oocysts (data not shown). This effect is trypsin specific, because the release of sporozoites was abolished when the soybean trypsin inhibitor was included in the incubation (data not shown).

These results indicated that the sporozoite egress from oocysts is a protease-dependent process and that CS-RIImut oocysts are more resistant to the trypsin treatment. CS is detected on the inner surface of the oocyst capsule [7–9]. Therefore, on their way out from the oocysts, sporozoites have to first traverse the CS protein layer beneath the capsule. The positively charged residues, arginines and lysines, of region II-plus are preferred substrates for certain cysteine proteases and serine proteases, such as trypsin. Therefore, it is possible that proteolysis of the CS protein layer underneath the capsule is required for sporozoite egress and is abolished in the mutant in which region II-plus has been substituted. Thus, a possible explanation for the defect of the mutant sporozoite is that the substitutions made in the region II-plus render CS protein more resistant to the putative protease. Trypsin treatment of the oocysts most likely cleaved the CS protein in many places, not only in region II-plus, since lysines and arginines are very abundant in the CS domains outside the repeats. Nevertheless, the subtle mutations introduced in CS region II-plus resulted in a clear difference in the kinetics of sporozoite release after treatment with the enzyme.

The exit of sporozoites from oocysts is most likely a stepwise process aimed at the sequential disruption of the capsule and the mosquito-derived basal lamina. In *P. falciparum,* PfCCp2 or PfCCp3, two secreted multidomain putative adhesive proteins, play an essential role in sporozoite release—in the absence of either protein, sporozoites were not released from the oocysts—but their localization and mechanism of action are unknown [22]. Our observations suggest that proteolysis of CS protein that lies beneath the capsule is likely to be an early event in sporozoite egress. Our hypothesis is supported by a recent finding that a papain-like cysteine protease egress cysteine protease 1 (ECP1) is required for sporozoite egress from oocysts [23]. Members of the papain family of cysteine proteases, similar to trypsin, consistently attack peptide bonds formed by lysine and arginine. We presume that ECP1 is only active when oocysts are “mature” and the sporozoites are ready to enter the hemolymph. At that particular time, only the CS protein that is beneath the capsule, but not CS on the sporozoite surface, is cleaved by ECP1, facilitating the egress of sporozoites from the oocysts. A possible explanation for this selectivity is that the enzyme that cleaves the capsule CS protein is also part of the capsule. We cannot exclude the possibility that the egress of sporozoites from oocysts is preceded by a proteolytic cascade and that ECP1 is only one of the participants.

### CS-RIImut Oocysts Sporozoites Are Not Infective to Mammalian Hosts

**Figure 5:**
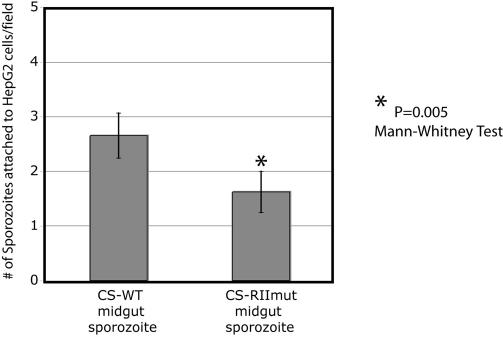
Region II-plus is Important for Sporozoite Adhesion to HepG2 Cells Midgut sporozoites of CS-WT and CS-RIImut (100,000 each) were added to confluent HepG2 cells. Adhesion is shown as the mean number of bound sporozoites in one microscopic field (400× magnification). Results are from three independent experiments.

As mentioned earlier, in vitro experiments strongly suggest that the region II-plus of CS protein plays an important role in the initial stages of sporozoite invasion of hepatocytes. Initial studies demonstrated that CS protein binds specifically to HSPGs in sections of human liver and that this binding is region II-plus dependent [24,25]. Synthetic peptides representing region II-plus specifically inhibit CS protein binding and sporozoite adhesion of HepG2 cells, the reference cell line that allows sporozoites to develop into mature exo-erythrocytic forms [26]. This inhibition is dependant on the downstream positively charged residues of region II-plus [26]. These and other findings (reviewed in [11,12]) give evidence that it is likely that the lysines and arginines (highly conserved in *Plasmodium* species) of region II-plus form ionic bonds with the negatively charged sulfate molecules of the HSPG glycosaminoglycan chains (GAGs). Our region II-plus mutant provided an opportunity to confirm the in vitro studies using a genetic approach. The wild-type and mutant sporozoites obtained by mechanical disruption of the midguts were incubated briefly with HepG2 cells to compare their binding to host cells. The sporozoites were obtained at a time when they are fully developed but not yet egressing from oocysts. There was a significant difference (~40%) in the binding of wild-type and mutant sporozoites (Figure 5). We emphasize that this assay was performed under static condition. The shear force generated by circulating blood in vivo should lead to more dramatic decrease in adhesion of mutant sporozoites to cells, as shown previously in vitro when the attachment assay was performed under rotating conditions [12]. Indeed, this is what we observed when we compared the infectivity of oocyst sporozoites from CS-WT and CS-RIImut to rats. The CS-WT and CS-RIImut sporozoites were injected intravenously into rats, and the pre-patent period of infection (time until the proportion of infected erythrocytes is less than 0.01%) measured. In two independent experiments, there was no infection in rats injected with 1–9 million CS-RIImut oocyst sporozoites (Table 2). In contrast, blood-stage parasites were detected in all rats injected with as few as 100,000 CS-WT oocyst sporozoites or 2,000 CS-WT salivary gland sporozoites [27].

**Table 2:**
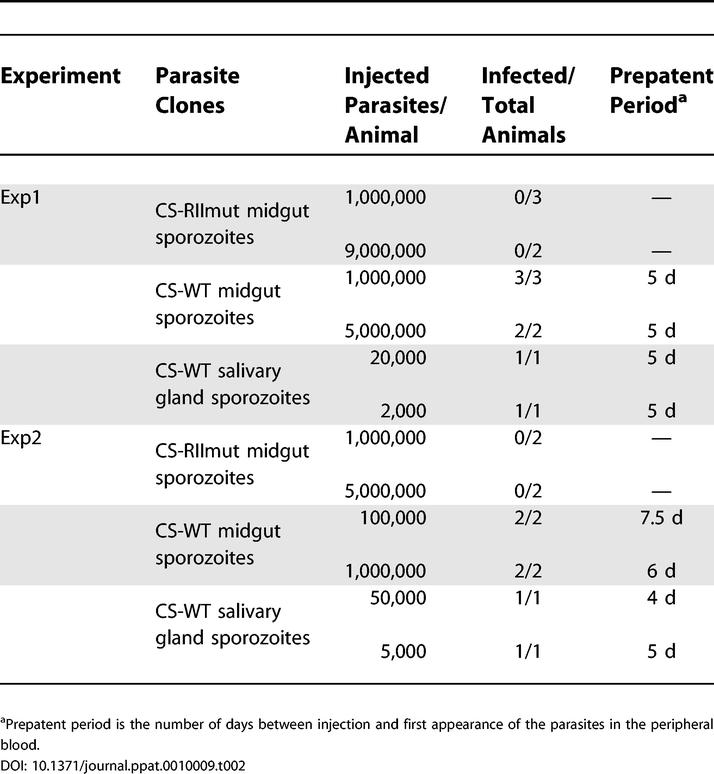
Infectivity of Sporozoites

Tewari et al. [28] also investigated the function of CS regions II-plus. In their study, the mutation was introduced in a *P. berghei* line in which the endogenous *CS (PbCS)* had been replaced by *P. falciparum CS (PfCS)*, leading to a substantial decrease in parasite infectivity [29,30]. In their studies, Tewari et al. deleted the entire region II-plus of CS protein, including two of the four cysteines of the CS thrombospondin domain, and noted that the mutants did not enter the salivary glands. However, they did not measure the number of parasites in the mosquito hemocoel. It is possible therefore that the Tewari's mutant has the same phenotype as ours.

We conclude that the same CS protein motif participates in two different stages of the sporozoite lifecycle. Region II-plus is first required for sporozoite egress from oocysts. Later it is required for the invasion of the mammalian hepatocytes. It is tempting to speculate that receptors for region II-plus are identical in the mammalian host liver and in the oocysts capsule/basal lamina in the mosquito midgut, i.e., they are HSPGs. The presence of HSPGs has been documented in *Drosophila* [31], and HSPG core proteins are represented in the *Anopheles* genome (http://www.ensembl.org) but have not yet been characterized biochemically. Perhaps capsule/basal lamina HSPGs interact with the positively charged stretch of amino acids of CS region II-plus, and the disruption of those peptide bonds by the newly identified cysteine protease (ECP1), or other participating proteases in the same proteolytic cascade, is an early and necessary step for sporozoite egress from oocysts.

## Materials and Methods

### Parasite.

The parasite is a wild-type pyrimethamine-sensitive, gametocyte-producing clone of the *P. berghei* NK65 strain.

### DNA construct and mutagenesis.

pQWCS-WT contains pUC19 backbone and 2.8 kb of CS cassette [32] cloned into XbaI and XhoI sites. Mutations were introduced into the CS coding region by using QuikChange Site-Directed Mutagenesis Kit (Stratagene, La Jolla, California, United States). Primer1 (sense, 5′-GGTATAAGAGTTGCTGCAGCAGCAGGTTCAAATAAGAAAGC-3′) and its reverse and complement primer2 were used to mutate R290, K291, R292, and K293 to alanines. The resulting construct is named “pQWCS-RIImut.”

### Targeting construct.

pMD205GFP is used as a backbone to generate targeting constructs, pRCS-WT and pRCS-RIImut. pMD205GFP contains the mutated copy of *P. berghei DHFR-TS* gene that confers resistance to pyrimethamine [33] and an *Aequorea victoria* green-fluorescent protein open reading frame (ORF) [34], and 2.2 kb and 0.55 kb of 5′- and 3′UTRs of *P. berghei* DHFR-TS. pQWCS-WT was digested with KpnI and XhoI to release the 2.1-kb fragment containing the CS cassette (0.6 kb of 5′UTR, 1-kb CS protein ORF and 0.5-kb 3′UTR). Subsequently, this fragment was cloned into pMD205GFP treated with same restriction enzymes to generate the intermediate construct pCS-WT. Targeting construct pRCS-WT was constructed by cloning a 0.6-kb BamHI-NotI fragment of CS 3′UTR (500–1,100 base pairs downstream of the stop codon) into the pCS-WT treated with the same restriction enzymes. Targeting construct pRCS-RIImut was constructed in the same way as pRCS-WT.

### Parasite transfection and genotype analysis.

Schizonts were collected for transfection, and targeting constructs were introduced by electroporation as previous described [33]. Southern blotting was performed with the entire CS ORF and 0.6-kb 5′UTR as a probe. The probe was labeled with DIG-ddUTP by random priming, and the chemiluminescence was detected using CSPD (Roche, Basel, Switzerland). Specific amplification of the 5′ recombinant locus was preformed with a forward primer CS1 (sense: 5′-CTTTTTCACCCTCAAGTTGGG-3′, which hybridizes to the CS 5′UTR missing in the pRCS-WT/pRCS-RIImut), and a reverse primer PB103 (sense, 5′-TAATTATATGTTATTTTATTTCCAC-3′, which hybridizes to the 5′UTR of DHFR-TS). Specific amplification of the 3′ recombinant locus was preformed with a forward primer PB106 (sense, 5′-TGTGCATGCACATGCATGTA-3′, which hybridizes to the 3′UTR of DHFR-TS), and a reverse primer CS4 (sense, 5′-CGAAATAAGTTACTATTCGTGCCC-3′, which hybridizes to the CS 3′UTR missing in the pRCS-WT/pRCS-RIImut).

### Mosquito infection and analysis of parasite development.

*A. stephensi* mosquitoes were fed on infected young Sprague-Dawley rats and dissected at various days PI. Midgut and salivary gland sporozoite populations were prepared from the various mosquito compartments and analyzed as previous described [27]. Hemolymph from each mosquito was perfused from the hemocoel with RPMI medium via air displacement from a micro-inoculation capillary inserted through the neck membrane and into the hemocoel. A small drop was made in the distal abdominal wall by gently removing the last two segments. The first three drops of perfusate (hemolymph and medium) from each mosquito were collected. Perfusate from at least 20 mosquitoes was collected. The number of sporozoites was determined using a haemocytometer.

### Indirect immunofluorescence assays.

Oocyst sporozoites were collected at day 18 PI, centrifuged onto glass slides, and fixed with 4% paraformaldehyde for 20 min at room temperature. Sporozoites then were pre-incubated in PBS-3% BSA for 1 h at 37 °C followed by incubation of various anti-CS antibodies for 1 h at 37 °C. Bound anti-CS was detected with FITC-conjugated anti-mouse IgG.

### Western blotting analysis of sporozoite lysates.

Protein samples were analyzed by SDS-PAGE and electrophoretically transferred to polyvinylidene difluoride membrane. CS-WT and CS-RIImut oocyst sporozoites on day 14 and 18 PI were collected, resuspended in SDS sample buffer, and incubated for 5 min at 70 °C prior to loading. The migrating bands were revealed with antibodies to *P. berghei* TRAP and CS protein, followed by horseradish peroxidase–coupled donkey anti-rabbit, and sheep anti-mouse IgG respectively, and visualized with enhanced chemiluminescence (ECL; Amersham Bioscience, Little Chalfont, United Kingdom).

### Analysis of sporozoite infectivity.

To analyze sporozoite motility, sporozoites were incubated in 3% BSA-RPMI 1640 medium for 3 h prior to microscopic examination [3]. To determine the infectivity of sporozoites in vivo, young Sprague/Dawley rats were injected intravenously with sporozoite suspensions in RPMI 1640. The parasitemia of inoculated rodents was checked daily by a 10-min examination of a Giemsa-stained blood smear.

### Sporozoite attachment assay.

A total of 100,000 midgut sporozoites were added and centrifuged down to confluent HepG2 cells. After 5-min incubation at 37 °C, cells were washed twice with PBS, and fixed with 4% formaldehyde. Adherent sporozoites were stained with a combination of anti-CS 3D11 and goat anti-mouse FITC antibodies. For each well, 25 microscopic fields were counted in duplicate using a 400× magnification.

### In vitro assay of oocyst sporozoite release.

Intact midguts were dissected from either CS-WT– or CS-RIImut–infected mosquitoes. For each experiment, 10 midguts were incubated at 25 °C in 200-μl RPMI medium with or without trypsin (50 μg/ml; Sigma, St. Louis, Missouri, United States). At different time points, 10 μl was taken out after gentle shaking the tubes, and sporozoites were counted. At the end of the experiment, the midguts were ground in order to determine the mean number of remaining oocyst sporozoites per mosquito.

### Transmission electron microscopy.

*P. berghei* (CS-WT and CS-RIImut) oocysts within mosquito midguts were fixed with 2.5% glutaraldehyde in 0.05 M phosphate buffer (pH 7.4) with 4% sucrose for 2 h. and then post-fixed in 1% osmium tetroxide for 1 h. After a 30-min en bloc stain with 1% aqueous uranyl acetate, the cells were dehydrated in ascending concentrations of ethanol and embedded in Epon 812. Ultrathin sections were stained with 2% uranyl acetate in 50% methanol and with lead citrate, and then examined in a Zeiss CEM902 electron microscope.

### Immunoelectron microscopy.

*P. berghei* oocysts within mosquito midguts were fixed with 3% paraformaldehyde, 0.25% glutaraldehyde in 0.1 M phosphate buffer (pH 7.4). Fixed samples were washed, dehydrated, and embedded in LR White resin (Polysciences, Warrington, Pennsylvania, United States) as described previously [7]. Thin sections were blocked in PBS containing 0.01% (v/v) Tween-20 and 5% (w/v) nonfat dry milk (PBTM). Grids were then incubated for 2 h at room temperature with the primary mouse anti-CS monoclonal antibody 3D11, and diluted 1:500 in PBTM. Normal mouse serum or PBTM were used as negative controls. After washing, grids were incubated for 1 h with 15-nm gold-conjugated goat anti-mouse IgG (Amersham Life Sciences), diluted 1:20 in PBS containing 1% (w/v) BSA and 0.01% (v/v) Tween-20, rinsed with Tween-20, and fixed with glutaraldehyde to stabilize the gold particles. Samples were stained with uranyl acetate and lead citrate, and then examined in a Zeiss CEM902 electron microscope.

## Abbreviations

CS: circumsporozoite
ECP1: egress cysteine protease 1
HSPG: heparan sulfate proteoglycan
ORF: open reading frame
PI: postinfection
TSR: thrombospondin type I repeats
